# Another functional frame-shift polymorphism of *DEFB126* (rs11467497) associated with male infertility

**DOI:** 10.1111/jcmm.12502

**Published:** 2015-02-27

**Authors:** Shiwei Duan, Changgeng Shi, Guowu Chen, Ju-fen Zheng, Bin Wu, Hua Diao, Lindan Ji, Yihua Gu, Aijie Xin, Yancheng Wu, Weijin Zhou, Maohua Miao, Limin Xu, Zheng Li, Yao Yuan, Peng Wang, Huijuan Shi

**Affiliations:** a Zhejiang Provincial Key Laboratory of Pathophysiology, School of Medicine, Ningbo UniversityNingbo, Zhejiang, China; b China National Population and Family Planning Key Laboratory of Contraceptive Drugs and Devices, Shanghai Institute of Planned Parenthood Research (SIPPR)Shanghai, China; c Obstetrics and Gynecology Hospital of Fudan University, Shanghai Jiai Genetics & In Vitro Fertilization (IVF) Institute Center of China-USAShanghai, China; d State Key Laboratory of Bioreactor Engineering, East China University of Science and TechnologyShanghai, China; e Shanghai Human Sperm Bank, Shanghai Institute of Andrology, Renji Hospital, Shanghai Jiao Tong University School of MedicineShanghai, China

**Keywords:** DEFB126, rs140685149, rs11467497, male infertility, indel, frame-shift

## Abstract

*DEFB126* rs140685149 mutation was shown to cause sperm dysfunction and subfertility. Indel rs11467497 is another 4-nucleotide frame-shift mutation (151bp upstream of rs140685149) that leads to the premature termination of translation and the expression of peptide truncated at the carboxyl terminus. In the present study, we performed a comprehensive association study to check the contribution of rs140685149 and rs11467497 to male infertility. Our results confirmed the previous findings that there was no association between rs140685149 and sperm motility. In contrast, we found a significant association of another indel rs11467497 with male infertility. Moreover, rs11467497 was shown to be associated with higher number of round cells in the infertile males with low sperm motility. Surprisingly, the two mutations commonly existed in the sperm donors (*n* = 672), suggesting a potential application of the two indels in the screening for eligible sperm donors. Western blotting assays showed the sperms with rs140685149 2-nt deletion tended to have unstable DEFB126 protein in contrast of no DEFB126 protein expressed in the sperms with rs11467497 4-nt deletion, suggesting a more severe consequence caused by rs11467497 mutation. In conclusion, our study presented a significant contribution of another functional frame-shift polymorphism of *DEFB126* (rs11467497) to male infertility.

## Introduction

Defensins are small microbicidal peptide toxins that are active against bacteria, fungi and enveloped viruses 1. Although the coding sequences of defensins are highly polymorphic, their protein products are conserved in structure. According to their size and disulphide binding patterns, mammalian defensins are classified into alpha-, beta- and theta-defensins. Alpha-defensins are primarily expressed in neutrophils as well as in NK cells and certain T lymphocyte subsets, regulating the microbial balance in the intestinal lumen 2. Beta-defensins are widely distributed on many organs including epididymis, implicating in the resistance of epithelial surfaces to microbial colonization. Beta-defensins code for genes which impact the function of the innate immune system 3. Theta-defensins do not naturally exist in humans. However, the artificial human theta-defensins can prevent viruses such as human immunodeficiency virus (HIV) from entering their target cells 4.

Approximately 15% of couples fail to attempt their first pregnancy. Male factor is at least partly responsible in about 50% of infertile couples. Among the many aspects, beta-defensins are found to play a primary role in the male infertility. Beta-defensin Defb42 mRNAs are found to be downregulated in mice model with male infertility 5. Disrupted expression of epididymal beta-defensins can inhibit sperm motility in rats 6. Being primarily expressed in epididymis 7, beta-defensin DEFB126 has been found to be one of the potential targets for the development of post-testicular male contraception 8. DEFB126 protein is secreted on the surface of sperm 9 through its binding on the lectins over the entire sperm surface 10. As a pore-forming glycopeptides, DEFB126 homodimer coats the immature spermatozoa in a cell-type specific manner until sperms become capacitated 11. DEFB126 glycopeptide is formed the entire surface of cynomolgus macaque sperm as they move through the corpus/caudal region of the epididymis 10. The coat of DEFB126 protects the sperm from the gram-negative bacterial infection 12 and the immune recognition in the female reproductive tract 13. Evidence has shown the coat of DEFB126 on the surface of sperm facilitates sperm penetration of cervical mucus and mediates attachment of sperm to oviductal epithelia 14. The loss of DEFB126 from sperm has implications for the timing of sperm release from the oviductal reservoir 15,16.

In the current study, we genotyped two *DEFB126* frame-shift indels among 2682 individuals from Shanghai Jiai Genetics & IVF Institute and Shanghai Human Sperm Bank. The goal of our study was to explore whether two common frame-shift indels of *DEFB126* gene contributed to the risk of male infertility in Han Chinese.

## Materials and methods

### Sample collection

As shown in Table S1, a total of 1361 infertile Chinese males with normal sperm counts (≥15 × 10^6^/ml) were recruited from Shanghai Jiai Genetics & IVF Institute. The infertile males consisted of 750 with normal sperm progressive motility (PR ≥ 40%) and 611 with much lower sperm motility (PR ≤ 20%). In addition, a total of 642 fertile males were recruited as controls from Shanghai Jiai Genetics & IVF Institute. We also obtained the blood samples of 679 Han Chinese males who donated their sperms in the Shanghai Human Sperm Bank. Genomic DNA was extracted from peripheral blood lymphocytes using DNA-isolation kits (Biovision Inc, Xiamen, China). All the individuals have signed the informed consent forms.

### Phenotyping

Sperm count and motility was measured by the computer-aided sperm analysis (CASA, Cyto-S, Alpha Innotech Corp. San Leandro, CA, U.S.A.) according to WHO laboratory manual for the examination and processing of human semen. The temperature of sperm process was maintained at 37°C.

### PCR and sequencing

According to the genomic DNA sequence of *DEFB126*, we used the primers (forward: 5′-TGTCTAAACGACGTTGGAATTT-3′; reverse: 5′-CCCTAGCATCACCTGGGAACT-3′) to amplify DNA fragment containing the two indels. PCR amplifications were carried out in a total volume of 50 μl buffered solution containing the primer mixture, 100 ng genomic DNA, 200 μmol/l deoxyribonucleotide triphosphates each, 1.5 mmol/l Mg^2+^ and 1.0 U Taq polymerase which was modified with anti-Taq antibody (Toyobo, Osaka, Japan). The cycling conditions were as follows: 95°C for 10 min., followed by 95°C for 10 sec., 57°C for 15 sec. and 72°C for 45 sec. for 40 cycles, a final extension at 72°C for 10 min. Products of the amplification were sequenced with sequencing primer (5′-AATTGGAAACTAAAAGTGAGCC-3′) provided by Biosun Limited Company (Shanghai, China).

### Genotyping

We used allele-specific PCR assay based on the SYBR Green to genotype the two indels of DEFB126 gene. The PCR cocktail solution contained 1.5 μl genomic DNA, 0.5 μl false-paired or right-paired primer, 5 μl Sharpvue 2× universal qPCR Master Mix (Biovue Technology, Shanghai, China) and 3 μl nuclease-free dH_2_O. The cycling conditions were as follows: 94°C for 10 min., followed by 94°C for 10 sec. and 60°C for 1 min. for 35 cycles. Based on the melting temperature shift genotyping assay, the loci of interest were genotyped. Our results showed there was 100% concordance between the results of DNA sequencing and the melting temperature shift genotyping. The sequences of the genotyping primers were shown in Table S2.

### Western blotting assay

Sperm cells were treated by the 10× lysis buffer (2% SDS, 100 mM Tris/HCl, pH 7.6) at 95°C for 3–5 min. DNA was then sheared by sonication to reduce the viscosity of the sample. The lysates were centrifuged at 16,000 × g for 5 min., and the supernatants were stored at −80°C. The concentration of protein extract was measured using BCA Protein Assay kit Thermo Fisher Scientific, Waltham, Massachusetts, USA. Protein samples (50 μg/lane) were analysed on 15% SDS-PAGE gels and transferred to polyvinylidene difluoride (PVDF) membranes Amersham Pharmacia Biotech with GE Healthcare, Little Chalfont, United Kingdom in a continuous buffer system at 20 V for 40 min. using semidry blotter (Bio-Rad Little Chalfont, United Kingdom). The blotted PVDF membrane was blocked with 1× NET (50 mM Tris-HCl pH 7.5, 150 mM NaCl, 0.1% NP-40, 1 mM ethylenediaminetetraacetic acid pH8.0, 0.25% gelatin) and the immune-detection was carried out using the ECL plus (ECL plus™) Western blotting detection system (GE Healthcare, UK Little Chalfont, United Kingdom.) with a 1:100 dilution of rabbit anti-DEFB126 polyclonal antibody (cat #sc-85535; Santa Cruz Biotechnology, Dallas, Texas, U.S.A.) and 1:1000 dilution of secondary antibody (HRP-conjugated Goat anti-Rabbit IgG).

### Statistical analyses

Tests of Hardy–Weinberg equilibrium (HWE) and genetic association on the genotype and allele levels were described in our previous studies 17–19. HWE test was done using the Arlequin program 20. Linkage disequilibrium (LD) and haplotypes frequencies were inferred using the Arlequin program based on the expectation-maximization algorithm 20. The inferred haplotype frequencies between two different groups were compared using the CLUMP22 software 21. Correlation test was analysed using the linear regression in the R is a free software programming language and software environment for statistical computing and graphics. We here provide a website (www.r-project.org) for its citation statistical software. A two-sided *P* < 0.05 was considered to be significant.

## Results

**Figure 1:**
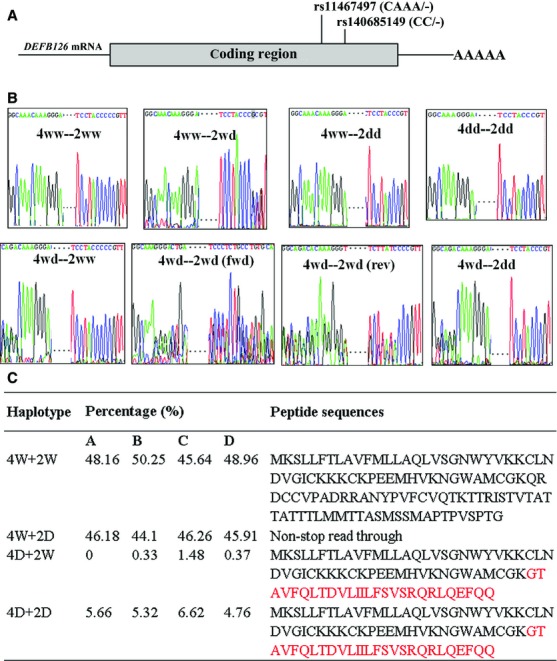
Interaction of the two indels of DEFB126 gene/a: 4ww denotes wide type of 4-nt indel (rs11467497); 4wd denotes rs11467497 ins/del genotype; 4dd denotes rs11467497 del/del genotype; 2ww denotes wide type of 2-nt indel (rs140685149); 2wd denotes rs140685149 ins/del genotype; 2dd denotes rs140685149 del/del genotype; 4W denotes rs11467497-ins allele; 4D denotes rs11467497-del allele; 2W denotes rs140685149-ins allele; 2D denotes rs1406-85149-del allele. (A) The locations of the two common indels on DEFB126 gene. (B) Sequencing plots of the two indels combinations. (C) Haplotype distribution of the two indels among the infertile males with normal sperm motility (group A), infertile males with low sperm motility (group B), fertile males (group C), and sperm donors (group D).

In the current study, we performed a comprehensive analysis of two *DEFB126* indels among four different groups, comprising 750 infertile males with normal sperm motility, 611 infertile males with low sperm motility, 679 healthy sperm donors and 642 fertile males (Fig.1, Table S1).

Our results showed that there was no departure of HWE for the two indels in the sperm donors (*P* > 0.05). However, there was a significant excess of heterozygotes for rs140685149 in the fertile males (observed = 413; expected = 320; χ^2^ = 54.32, df = 1, *P* < 0.001), implying an advantage for the heterozygotes of rs140685149 in the fertile males. This agreed to the previous observation 22. No significant association was observed between the two indels (rs140685149 and rs11467497) and sperm characteristics including sperm concentration and motility (*P* > 0.05). This observation also met with the previous findings 22. Our results implied that DEFB126 coat on sperm surface might be not related to sperm production and motility.

**Figure 2:**
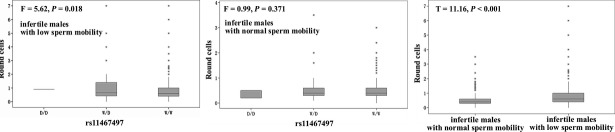
Round cells and rs11467497 in the infertile males with low and normal sperm motility.

Surprisingly, we observed a significant association of another indel rs11467497 with male infertility on both genotype and allele levels (Table1, fertile males *versus* infertile males with normal sperm motility: χ^2^ = 6.42, df = 1, *P* = 0.01; fertile males *versus* infertile males with low sperm motility: χ^2^ = 5.8, df = 1, *P* = 0.01). We also observed significant contribution of rs11467497-del to male infertility under the dominant inheritance model (Table2, fertile males *versus* infertile males with normal sperm motility: OR = 0.65, 95% CI = 0.47–0.89, *P* = 0.007; fertile males *versus* infertile males with low sperm motility: OR = 0.66, 95% CI = 0.48–0.92, *P* = 0.014). Not surprisingly, much higher number of round cells was observed in the infertile males with low sperm motility than in the infertile males with normal sperm motility (T = 11.16, *P* < 0.001). In addition, rs11467497-del was significantly associated with higher number of round cells in the infertile males with low sperm motility (Fig.2, F = 5.62, *P* = 0.018), but not in the infertile males with normal sperm motility (Fig.2, *P* > 0.05).

**Table 1:** Genotype and allele analysis of the two common indels of *DEFB126* gene*

	Genotype (counts)					Allele (counts)			
	WW	WD	DD	χ^2^ (*P*)†	χ^2^ (*P*)‡	W	D	χ^2^ (*P*)†	χ^2^ (*P*)‡
rs140685149									
Infertile males I	162	402	186			726	774		
Infertile males II	150	314	147			614	608		
Fertile males	96	413	133	17.56 (<0.001)	25.28 (<0.001)	605	679	0.46 (0.51)	2.45 (0.12)
Sperm donors	171	333	175	3.54 (0.18)	0.78 (0.68)	675	683	0.49 (0.50)	0.08 (0.82)
rs11467497									
Infertile males I	653	77	3			1383	83		
Infertile males II	535	66	1			1136	68		
Fertile males	540	100	2	7.90 (0.014)	6.04 (0.037)	1180	104	6.42 (0.01)	5.80 (0.01)
Sperm donors	604	67	1	0.96 (0.69)	0.34 (0.78)	1275	69	0.38 (0.56)	0.33 (0.59)

* Infertile males I group refers to the infertile males with normal sperm counts and motility. Infertile males II group refers to the infertile males with normal sperm count but low motility.

† The comparison between Infertile males I group and the corresponding group.

‡ The comparison between Infertile males II group and the corresponding group.

**Table 2:** Association between the two indels of *DEFB126* gene and male infertility under dominant models*

Dominant model	(DD+WD)/WW	OR (95% CI)†	*P* (df = 1)†	OR (95% CI)‡	*P* (df = 1)‡
rs140685149					
Infertile males I	598/162				
Infertile males II	461/150				
Fertile males	546/96	0.65 (0.49–0.86)	0.002	0.54 (0.41–0.72)	<0.001
Sperm donors	508/171	1.24 (0.97–1.59)	0.08	1.04 (0.80–1.33)	0.79
frs11467497					
Infertile males I	80/653				
Infertile males II	67/535				
Fertile males	102/540	0.65 (0.47–0.89)	0.007	0.66 (0.48–0.92)	0.014
Sperm donors	68/604	1.09 (0.77–1.53)	0.62	1.11 (0.78–1.59)	0.56

* Infertile males I group refers to the infertile males with normal sperm counts and motility. Infertile males II group refers to the infertile males with normal sperm count but low motility.

† The comparison between Infertile males I group and the corresponding group.

‡ The comparison between Infertile males II group and the corresponding group.

A LD test of the two indels showed that they were in high LD but not in high correlation (Table S3), although these two indels were only 151bp away from each other (Fig.1). In addition, we also observed a significant contribution of rs140685149(ins)-rs11467497(del) haplotype to male infertility (Table3).

**Table 3:** Comparison of estimated haplotypes between the infertile males and the control groups*

rs11467497	rs140685149	Infertile male I	Infertile male II	Fertile males	Sperm donors	Pa, OR (95% CI)	Pb, OR (95% CI)	Pc, OR (95% CI)	Pd, OR (95% CI)
W	W	706	605	586	658	0.19, 1.11 (0.95–1.29)	0.67, 0.97 (0.84–1.12)	0.02, 1.20 (1.03–1.41)	0.52, 1.05 (0.91–1.23)
W	D	677	531	594	617	1.00, 1.00 (0.86–1.16)	0.89, 1.01 (0.87–1.17)	0.28, 0.92 (0.78–1.07)	0.36, 0.93 (0.80–1.09)
D	W	0	4	19	5	NA, NA	NA, NA	0.003, 0.22 (0.08–0.65)	1, 0.89 (0.24–3.33)
D	D	83	64	85	64	0.29, 0.85 (0.62–1.16)	0.29, 1.20 (0.86–1.68)	0.17, 0.79 (0.57–1.11)	0.52, 1.12 (0.79–1.60)

* NA denotes not analysed; Pa were calculated between Infertile males I and Fertile males; Pb were calculated between Infertile males I and Sperm donors; Pc were calculated between Infertile males II and Fertile males; Pd were calculated between Infertile males II and Sperm donors.

**Figure 3:**
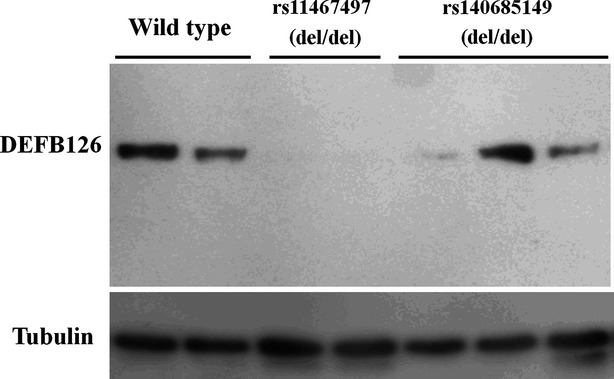
DEFB126 expression in sperms with various DEFB126 genotypes.

According to the results of the Western blotting assays, sperms with rs11467497 del/del genotype were unable to express DEFB126 protein, in contrast that the sperms with rs140685149 del/del genotype expressed DEFB126 protein at variable levels (Fig.3). These results suggested rs11467497 caused a more severe effect on DEFB126 function than rs140685149 did.

## Discussion

In the present study, we investigated the two *DEFB126* frame-shift indels in four groups of individuals including infertile males with normal sperm phenotypes, infertile males with low sperm motility, fertile males, and healthy sperm donors. To our surprise, we found a 4-nt frame-shift indel (rs11467497) was found to be associated with male infertility, although it was only 151bp away from rs140685149, 2-nt frame-shift indel. Moreover, these two indels were commonly found in the sperm donors, suggesting a potential application of these markers in screening for eligible sperm donors in the future. In addition, our results confirmed that *DEFB126* mutations were not related to sperm production and motility. We also showed there was a significant excess of rs140685149 heterozygote genotype in the fertile males, suggesting a selective advantage of this mutation.

However, another 4-nt indel (rs11467497) was found to be associated with the protection of male infertility. This 4-nt indel (rs11467497, CAAA/-) has been found in multiple HapMap populations (including Japanese in Tokyo, Japan, Han Chinese in Beijing, China, Utah residents with ancestry from northern and western Europe and Yoruba in Ibadan, Nigeria) as reported in the 1000 genomes project 23. This 4-nt deletion genotype causes a premature termination of translation and the expression of proteins truncated at the carboxyl terminus (Fig.1). Meanwhile, it was also shown to be associated with a higher number of round cells in the infertile males with low sperm motility (F = 5.62, *P* = 0.018, Fig.2). The round cells consist of spermatogenic and non-spermatogenic round cells in semen. The presence of neutrophils in the non-spermatogenic round cells often indicates an infection and/or a subsequent inflammatory reaction in the male genital tract 24 that may have influence on male fertility 25–27. Leukocytic concentration was shown to be negatively associated with sperm morphological defects and sperm motility 28. In the current study, the association of the indel rs11467497 with round cell number was found only in the low-sperm-motility infertile males, suggesting a role of rs11467497 in the sperm morphological defects and motility. DEFB126 is a family member of antimicrobial peptides, and our data showed that the 4-nt deletion of *DEFB126* gene diminished the expression of DEFB126 in the sperms (Fig.3). We speculated that the loss of DEFB126 led to the occurrence of the round cells in the infertile males with *DEFB126* rs11467497 mutation. In summary, our findings provided new hints of DEFB126 in the pathogenesis and possibly treatment of human infertility 22,29–31.

This dinucleotide indel (rs140685149, CC/-) of *DEFB126* gene was found to affect its lectin binding property, thereby causing a defective sperm penetration 22. This indel was found to reduce male fertility substantially by impairing the penetrating function of sperms 32. Tollner and colleagues found that there were 19% Chinese husbands with rs140685149-del homozygote and 51% with rs140685149-in/del heterozygote among 638 newly-wed couples 32. There was a correlation between the deletion and the fertility of husbands 32. Functional analysis found that there was a drop of ability to penetrate the HA 32. However, this indel was shown to be not associated with the quality of sperm including sperm morphology and a series of CASA motion parameters among 16 samples 32. Moreover, there is an observation of departure of HWE of rs140685149 in the Tollner's study 32. Our data found a similar allele and genotype distribution in a larger set of samples and confirmed that rs140685149 was not associated with sperm motility. Interestingly, we also observed a significant excess of rs140685149 in/del heterozygote in the fertile males (*P* < 0.001). This suggests that there is likely to have a selective advantage for rs140685149 in/del heterozygote, although further work needs to reveal the underlying mechanism of this observation.

According to an online gene expression dataset (GSE740) 33 in the NCBI website, at least 7 defensin genes (*DEFB126*, *DEFB103A*, *DEFA4*, *DEFB1*, *DEFB4A*, *DEFA5* and *DEFA6*) are expressed in epididymis. Among these defensins, the expression level of *DEFB126* gene is 30 times of *DEFB103A* gene, and 40 times of *DEFA4* gene, and 48 times of *DEFB1* gene, and much higher than the rest defensin genes (*DEFB4A*, *DEFA5* and *DEFA6*). Interestingly, DEFB126 were down regulated in the caput epididymides of non-obstructive azoospermic men 34. All these implied a predominant role of *DEFB126* gene in the sperm activity in the epididymis.

DEFB126 protein has a conserved β-defensin core and a unique long glycosylated peptide tail. The carbohydrates of this domain contribute substantially to the sperm glycocalyx. Although DEFB126 protein is conserved in structure DEFB126 gene is highly polymorphic according to the records in the NCBI dbSNP database. Altogether there are 77 known SNPs in the gene region, including 44 intronic SNPs, 11 synonymous SNPs, 18 missense SNPs, 6 frame-shift SNPs (rs200807952 (-/GCAA); rs11467497 (-/CAAA); rs74380987 (CAAA/-/C); rs11469647 (CAAA/-); rs11467417 (-/CC/C); rs140685149 (-/CC)) and 1 UTR SNP. As only two frame-shift SNPs (rs140685149, rs11467497) were involved in the present study, we cannot exclude the possibility that other *DEFB126* SNPs contribute to the male infertility. Future analyses of other genetic variants or epigenetic modifications may help elaborate the contribution of DEFB126 gene to the male infertility.

The sperm surface proteomics has enabled researchers to search for the coverage of the proteins at the sperm surface that may help understand the milieu of the sperm cell during transit from the testis to the oviduct 35,36. Besides the influence of DEFB126 on the sperm surface, there are other sperm surface membrane proteins (such as ACE, HSPA4L, *etc*.) that may be important for the sperm migration to oviduct 36. Searching for functional variants of these genes is likely to become the direction of future research on male infertility.

In conclusion, we performed a large scale genetic testing of two functional mutations of *DEFB126* gene for the association with male infertility. Our results showed that rs11467497-del was associated with male infertility and higher number of round cells in the infertile males with low sperm motility. We also found a lack of association between rs140685149 and sperm motility through the comparison between two types of infertile males with different sperm motility. Further work is needed to elucidate the selection advantage of rs140685149 del/ins heterozygotes in the fertile males.

## Supporting Information

**Table S1** Four groups of samples involved in the current study. **Table S2** Genotyping primers of the two indels of DEFB126 gene. **Table S3** LD between the two indels of DEFB126 gene. **Data S1** The sequences of seven samples with different combination of 2 indels.
