# Supplementary material for: Another functional frame-shift polymorphism of DEFB126 (rs11467497) associated with male infertility
Source: J Cell Mol Med. 2015 Feb 27;19(5):1077–84. doi: 10.1111/jcmm.12502 (PMC4420609; doi:10.1111/jcmm.12502)

Supplemental Table 1: Four groups of samples involved in the current study

| Groups | number |
| --- | --- |
| Infertile Han Chinese males with normal sperm motility | 750 |
| Infertile Han Chinese males with low sperm motility | 611 |
| Fertile males | 642 |
| Han Chinese males who have donated their sperms | 679 |

Supplemental Table 2: Genotyping primers of the two indels of *DEFB126* gene

| ID | Genotype^a^ | Forward | Reverse | Length (bp) |
| --- | --- | --- | --- | --- |
| rs1146837 | WW | AAGGGACTGCTGTGTTCCAG | ACCAGTGGGAGAA  ACGGGCGT | 169 |
|  | DD | CTTCGATGGCTCCTACGCG | GCTGTGGGCCTAG  AACTGTC | 295 |
| rs11467497 | WW | GCCCCTGGTAAGTGTTTGTA | CACAGCAGTCCCT  TTGTTAGC | 366 |
|  | DD | GCAATGTGCGGCAATGG | CAGGAGACAGAG  GCTGGAAT | 207 |

a: WW denotes insertion homozygotes; DD denotes deletion homozygotes

Supplemental Table 3: LD between the two indels of *DEFB126* gene

| Groups |  | rs140685149 | rs11467497 |
| --- | --- | --- | --- |
| Infertile males I | rs140685149 | — | **0.056** |
|  | rs11467497 | *1* | — |
|  |  |  |  |
| Infertile males II | rs140685149 | — | **0.049** |
|  | rs11467497 | *0.9* | — |
|  |  |  |  |
| Fertile males | rs140685149 | — | **0.029** |
|  | rs11467497 | *0.6* | — |
|  |  |  |  |
| Sperm donors | rs140685149 | — | **0.038** |
|  | rs11467497 | *0.9* | — |

Numbers in bold are r^2^ values (upper diagonal), numbers in italic are D’ values (lower diagonal).

Supplemental material 1: The sequences of seven samples with different combination of 2 indels.

1) 4WW-2WW（4183#）


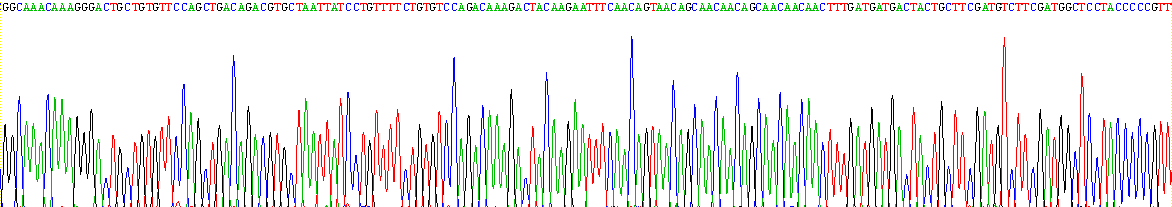


2) 4WW-2DD（4185#）


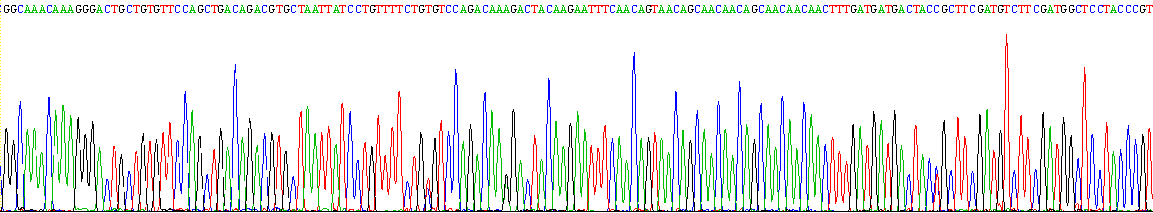


3) 4DD-2DD（4184#）


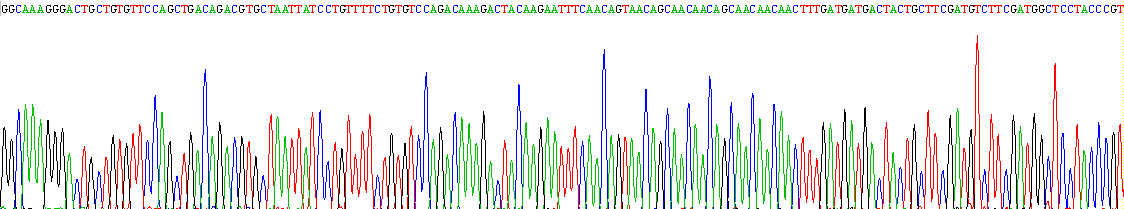


4) 4WW-2WD（4287#）


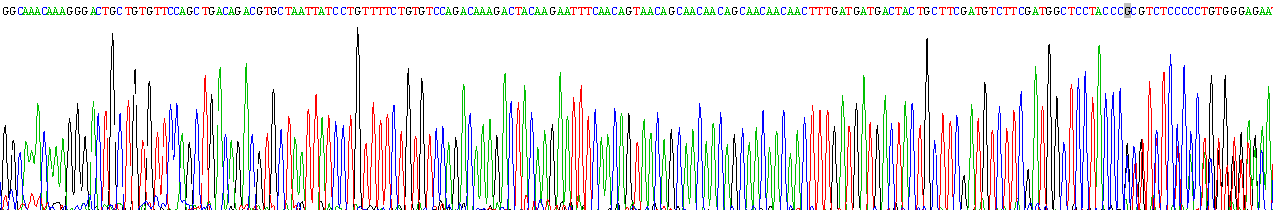


5) 4WD-2DD（3606#）


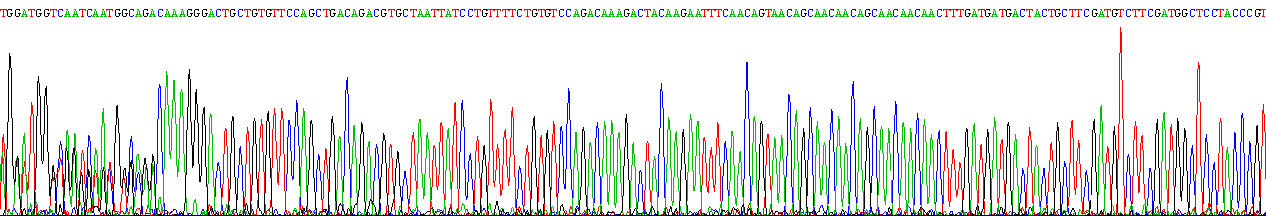


6) 4WD-2WW（820#）


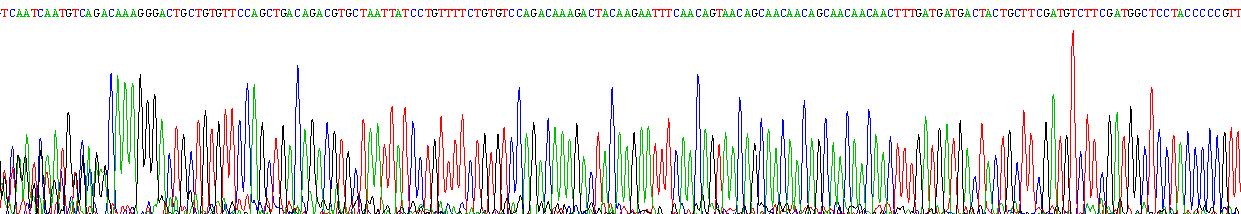


7A) 4WD-2WD_FWD（4282#）


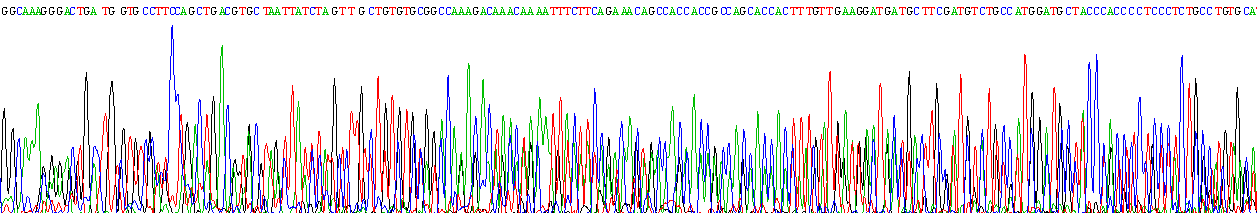


7B) 4WD-2WD_REV（4282#）


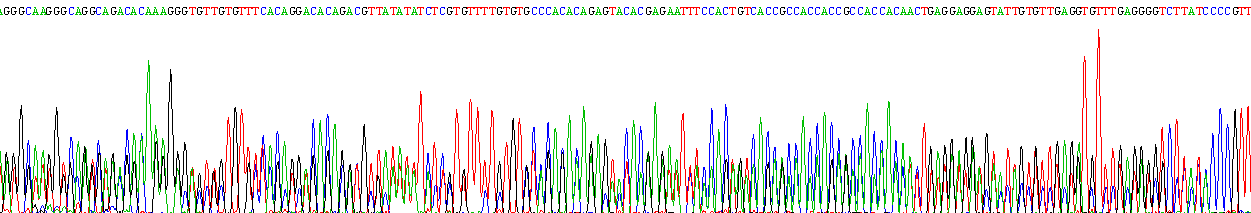

Supplement: Supplementary file 1 [file jcmm0019-1077-sd1.doc]
